# The Use of Sulphate of Cinchonidia in Acute Bright’s Disease and in Intermittent Fever

**Published:** 1878-07-20

**Authors:** J. C. McMechan

**Affiliations:** Physician to St. Mary’s Hospital, Cincinnati


### THE USE OF SULPHATE OF CINCHONIDIA IN ACUTE BRIGHT'S DISEASE AND IN INTERMITTENT FEVER.

By J. C. McMechan, M.D., Physician to St. Mary's Hospital, Cincinnati.

On September 21, 1877, I was consulted by J. A., of Maude Station, Ohio, who was suffering from intermittent fever. The usual remedies for that disease were prescribed, and in fourteen days the patient was well of the fever. A few days after the fever was cured, the patient's lower extremities began to swell, and became œdematous, and in a short time, they were so much swollen that J. H. was scarcely able to draw on his pantaloons. At this time, he consulted me again, and, upon examining his urine, I found it loaded with albumen. From the œdema present, and the albumen found in the urine, and no other disease being present, I considered the case to be one of acute Bright's disease. I advised the patient to go to the St. Mary's Hospital, and he entered that The patient was twenty-six years of age, tall and well developed, and, during his whole life, had never been sick until this attack. The first two weeks he was in the hospital, he took small doses of elaterium, and he also took tincture of digitalis. With this treatment, he improved but slowly. The œdema was not so well marked, but the amount of albumen in the urine was not diminished. About the first of November, he began to take thirty grains of sulphate of cinchonidia, daily. This was given in ten-grain doses three times per day. At the same time, he took 25 drops of tincture muriate of iron four times per day. Small doses of elaterium were also administered occasionally. After pursuing this course of treatment for two or three weeks, marked improvement was noticed; the œdema began to disappear, and the amount of albumen in the urine began to diminish. From this time, only twenty grains of cinchonidia were given daily, and the elaterium was given but seldom. About December 15, the œdema disappeared completely, and no trace of albumen was to be found in the urine.

About January 1, the patient was discharged from the hospital, and since then, there has been no return of the disease.

In February, 1878, Willie M., aged nine years, came under my care. He had been affected with dropsy for nine months previous to February, 1878. His father and mother were not able to state what was the beginning or cause of his sickness, but they were able to state that the dropsy had existed for nine months. His urine was found very albuminous, and no heart lesion or other diseased condition was found to exist. The case was diogrosticated acute Bright's disease, and the patient at once put upon five-grain doses of cinchonidia three times per day, and 20 drops of tincture ferri muriat. three times per day. Pills, containing small doses of elaterium, were also prescribed. The pills were to be taken but once a week. After three weeks of this treatment, the dropsy had almost disappeared, and the albumen was rapidly diminishing in amount. The patient has gradually been improving, and about two weeks ago, upon examining the urine, but a slight trace of albumen was found. The dropsy and œdema had disappeared. I think this last patient, like the first one, will recover completely. I have two other patients under my care affected with Bright's disease, the one case being acute and the other chronic. They have not been long enough in the hospital to test the effects of the conchonidia treatment fully, and I have no hopes of the chronic case being benefited. As suggested by an article in the *Clinic*, the cinchonidia acts well in cases of acute Bright's disease, following intermittent fever, and I have no doubt but it will act well in cases occurring after other diseases.

From September, 1877, to May 1, 1878, I have treated 56 cases of intermittent fever, in the wards of St. Mary's Hospital, with conchonidia. Of these 56 cases, only two cases had any return of the fever after undergoing a short course of treatment. Some of the patients had been affected with the fever for six months, and some for a year. One patient had contracted the disease in the South, while living in a swampy region, and came North for the express purpose of undergoing treatment, as he could get no relief from the remedies taken in the South. Several of the cases were from the malarious districts of Indiana. The usual dose of the cinchonidia given was twenty grains, divided into two doses, to be taken four and three hours before the time for the chill. In all of the severe cases, Fowler's solution and tinct. ferri muriat. were administered in conjunction with the cinchonidia. In only one case, 40 grains were administered during the twenty-four hours, and in only a few cases, 30 grains of cinchonidia were given during twenty-four hours. I never had better success in curing intermittent fever with quinia than I have had with the cinchonidia. In the hospital, this drug is administered in wafers, ten grains being placed in each wafer. In private practice, it is pleasanter to give the drug in capsules, each one to contain five grains. A very small capsule can be used if the druggist first moistens the cinchonidia with amylum glycerine before putting it in the capsules. Dr. Sidney Spence, of St. Bernard, Ohio, informs me that he uses nothing but cinchonidia in treating cases of intermittent fever, and he always succeeds in curing his cases as rapidly as he formerly did with quinia.

I am, at present, testing the effects of sulphate of cinchona in cases of intermittent fever.

In conclusion, I would say that, in my opinion, the sulphate of cinchonidia is just as effective as quinia—it looks like quinia, it has the same bitter taste that quinia has, it causes tinnitus aurium in the same doses that the latter drug does, and it requires no larger doses of it to cure the fever.—*Clinic.*

In connection with sulphate of cinchonidia, we take occasion to refer to cinchonia alkaloid, and the preparation from it of a tasteless anti-periodic. Among a number of articles that have been written in regard to the preparation of a tasteless mixture was one which appeared in the February number of the *American Journal of Pharmacy*, (1871), by Dr. J. B. R. Purnell, from which we quote the following:

"In the case of anything employed to conceal the taste of quinia sulphate, and like bitters, use the bitter in powder, avoiding an acid, or (with a few exceptions) any perfect solution."

